# Colon metastasis from hepatocellular carcinoma: a case report and literature review

**DOI:** 10.1186/s12957-020-01960-2

**Published:** 2020-07-28

**Authors:** Yong-Ming Yu, Yi-Sheng Cao, Zhou Wu, Rong Huang, Zhong-Lei Shen

**Affiliations:** 1 Department of General Surgery, HwaMei Hospital, University of Chinese Academy of Sciences, Ningbo, 315000 China; 2 Ningbo Institute of Life and Health Industry, University of Chinese Academy of Sciences, Ningbo, 315000 China; 3 Key Laboratory of Diagnosis and Treatment of Digestive System Tumors of Zhejiang Province, Ningbo, 315000 China; 4 Ningbo Pathological Diagnosis Center, Ningbo, 315000 China

## Abstract

**Background:**

Hepatocellular carcinoma (HCC) is a malignant tumor with frequent intrahepatic metastases; extrahepatic metastases are not rare but less frequent compared to intrahepatic ones. The most frequent sites of extrahepatic metastases are the lungs, followed by the lymph nodes, bones, and adrenal glands. Case report covering gastrointestinal (GI) tract involvement from HCC is limited.

**Case presentation:**

A 60-year-old man was referred to us in May 2019 with a diagnosis of sigmoid colon tumor. The patient had a history of HCC and had received two stages of open resections for the primary and the abdominal metastasis successively and many times of transcatheter arterial chemoembolization (TACE). The sigmoid colon tumor received Hartmann procedure after abdominal enhanced computerized tomography (CT) scan and colonoscopy, while postoperative pathology and immunohistochemistry identified it as extrahepatic colonic metastasis from HCC.

**Conclusions:**

The ratio of extrahepatic metastasis to the digestive tract was very low, and the majority was upper gastrointestinal involvement because of direct invasion or intraperitoneal implantation. TACE may be the risk factor of retrograde hematogenous metastasis to the downstream colon.

**Keywords:**

Hepatocellular carcinoma; Extrahepatic metastases; Colon metastasis; Transcatheter arterial chemoembolization

## Background

Hepatocellular carcinoma (HCC) is a highly malignant tumor all over the world and is also a global disease with high mortality. With the largest population of hepatitis B infections, China has the greatest number of primary liver cancer, and the incidence rate is 26.67/100,000; that is over 373,000 new cases per year, of which 80% are HCC [1]. Extrahepatic metastases are less frequent than intrahepatic ones with the incidence reported between 13.5–42% of metastasis cases [2]. The most frequent extrahepatic metastatic sites are the lungs, followed by the lymph nodes, bones, and adrenal glands, while gastrointestinal (GI) tract involvement is rare among these extrahepatic metastases. Case report covering GI involvement from HCC was limited and in lack of comprehensive analysis. We report a rare case of extrahepatic metastasis of the colon from HCC and review the related literature on the disease.

## Case presentation

A 60-year-old man, who had hematochezia for 45 days, was referred to our department of colorectal surgery on May 15, 2019. He was diagnosed with sigmoid colon tumor after colonoscopy showed a protuberant mass occupying the lumen with stenosis, 30 cm proximal to the anus. The patient had a history of hepatitis B since 1979 and was treated by Traditional Chinese Medicine (TCM) only. He was first rushed to hospital in emergency in 2009 with the complaint of right upper abdominal pain for 7 h. After a CT scan, he was confirmed to have the rupture and hemorrhage due to liver cancer, as well as cirrhosis and splenomegaly. Right hepatectomy was performed, and postoperative pathology showed a moderately differentiated hepatocellular carcinoma 65 × 31 × 61 mm in size with nodular cirrhosis, but the status of the lymphatic or vascular involvement was not reported by then. After the surgery, entecavir was prescribed by a hepatologist to control the hepatitis B virus (HBV) infection. In October 2010, intrahepatic nodules were found during regular follow-up and treated by interventional therapy. In April 2013, arterial blood supply appeared in two nodules near the diaphragmatic surface of the right lobe of the liver when doing magnetic resonance imaging (MRI) follow-up, and transcatheter arterial chemoembolization (TACE) was used. In August 2013, anti-HBV treatment was restarted when HBV-DNA copy soared to 3.46 × 10^5^ IU/ml. For the coming four and a half years, no signs of recurrence or metastasis was found until February 2018, when the patient felt a growing mass on the left side of the abdomen, and his alpha-fetoprotein (AFP) level went up to as high as 5.00 × 10^4^ ng/ml. CT scans considered the mass in his left middle abdomen to be an intraperitoneal implantation metastasis, 200 × 150 mm in size, and MRI also showed an intrahepatic metastasis in the infero-posterior segment of the right lobe of the liver. TACE was again implemented to treat intrahepatic metastasis followed by abdominal tumor resection. HCC with moderate and low differentiation associated with hemorrhage and necrosis was the pathological diagnosis. Immunohistochemistry revealed positive expression of AFP and hepatocyte, and negative in carcinoembryonic antigen (CEA). In May 2018, 3 months after the surgery, another intrahepatic metastasis was detected by MRI. In order to eliminate the focus and any possible micro-metastasis, radiofrequency ablation and TACE were performed successively in the case of adequate liver function reservation. In November 2018, the patient’s AFP level gradually rose to 2.91 × 10^3^ ng/ml, and MRI showed enhanced blood supply to the subcapsular lesion at the upper posterior segment of the right lobe of the liver, which was considered to be a recurrence. Ultrasound-guided percutaneous radiofrequency ablation (PRFA) was carried out, as well as TACE 3 weeks after that. Recovering from those therapies, the patient began to take Sorafenat (400 mg, twice a day) as targeted therapy for 4 months, and then replaced by regorafenib (160 mg, once a day, last for 3 weeks, every 4 weeks repeat) at the end of March 2019 because of continuously rising AFP level. Programmed cell death protein 1 (PD-1) inhibitor toripalimab injection was added as an immunotherapy in April 2019.

**Fig. 1:**
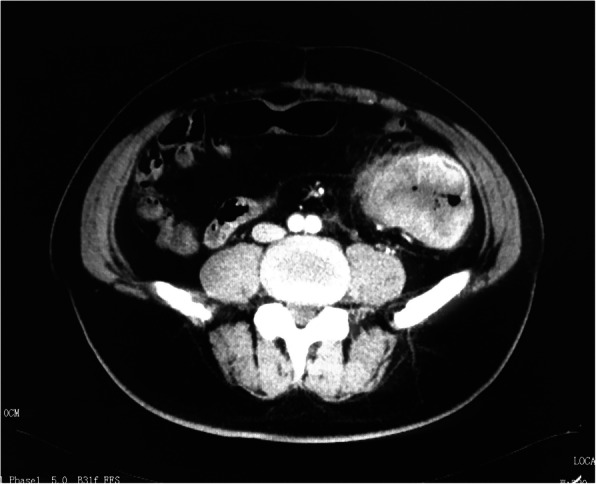
A huge tumor located in sigmoid colon with enhanced thickened bowel wall

**Fig. 2:**
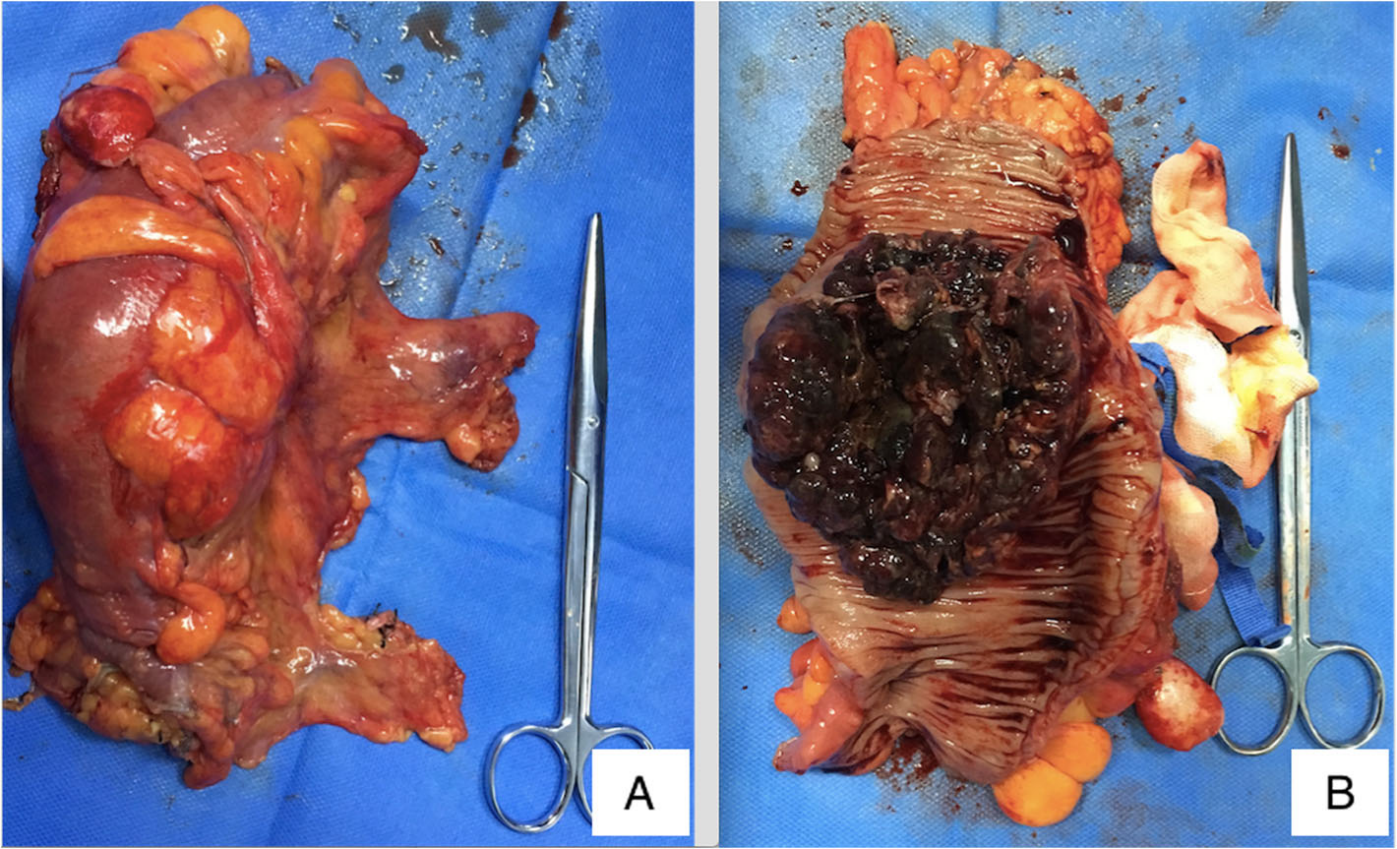
A large endogenic mass of the colon (**a**) with relatively smooth serous layer (**b**)

**Fig. 3:**
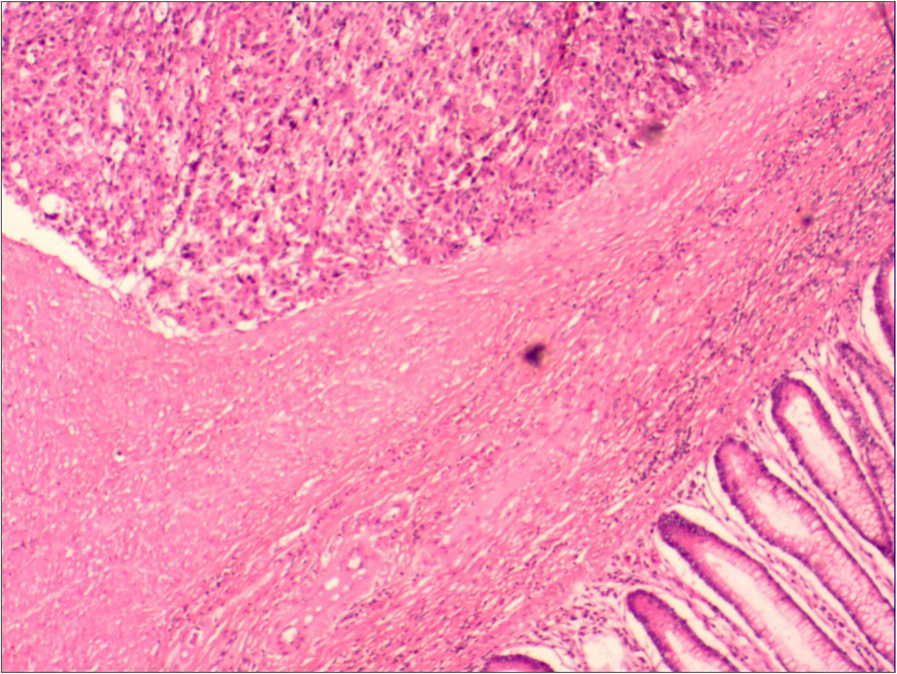
The neoplastic components (upper left) gathering in muscularis propria without transition from the intestinal mucosal epithelium (lower right) (HE stain, 4 × 10)

**Fig. 4:**
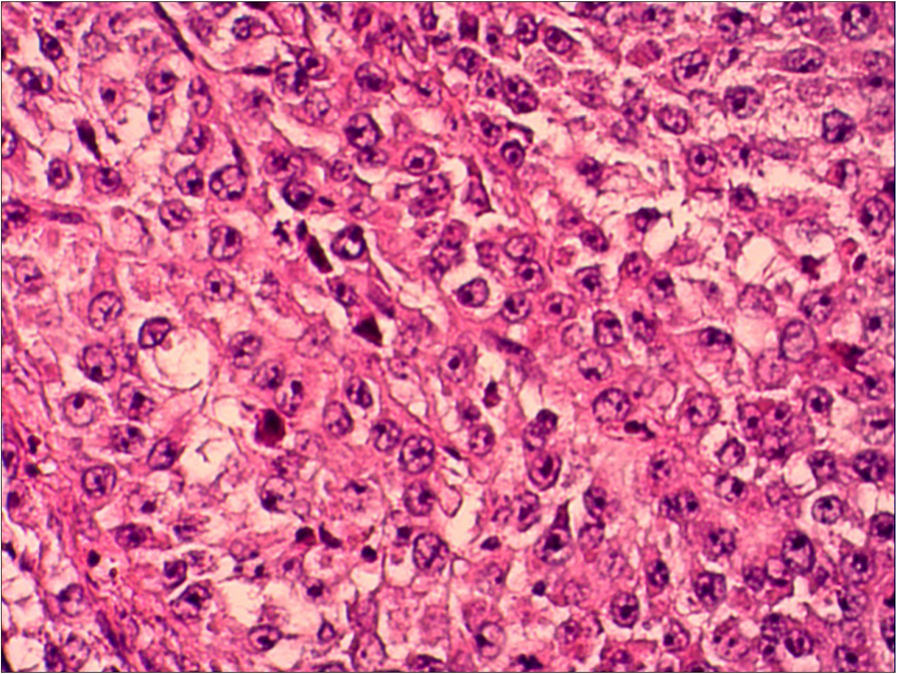
The neoplastic cells are arranged in nests, and the nucleoli are clearly visible. Neoplastic giant cells and nuclear division can be seen, suggesting poor differentiation (HE stain, 20 × 10)

**Fig. 5:**
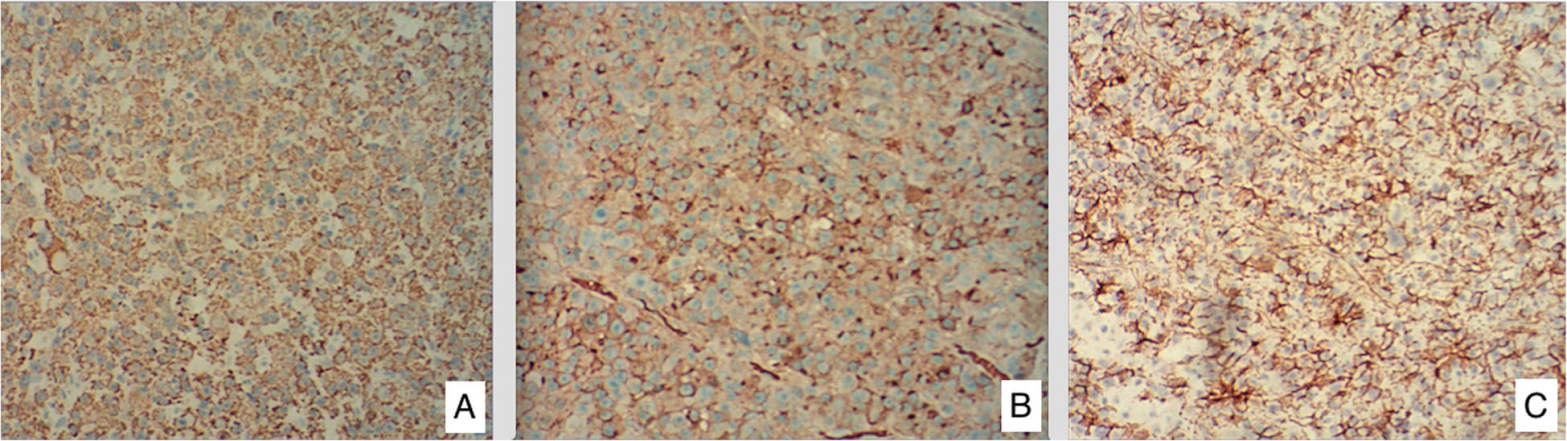
Immunohistochemical stain reveals positive marking result in HSP70 (**a**), AFP (**b**), and B-catenin (**c**), supporting hepatogenic or hepatoid differentiation

**Fig. 6:**
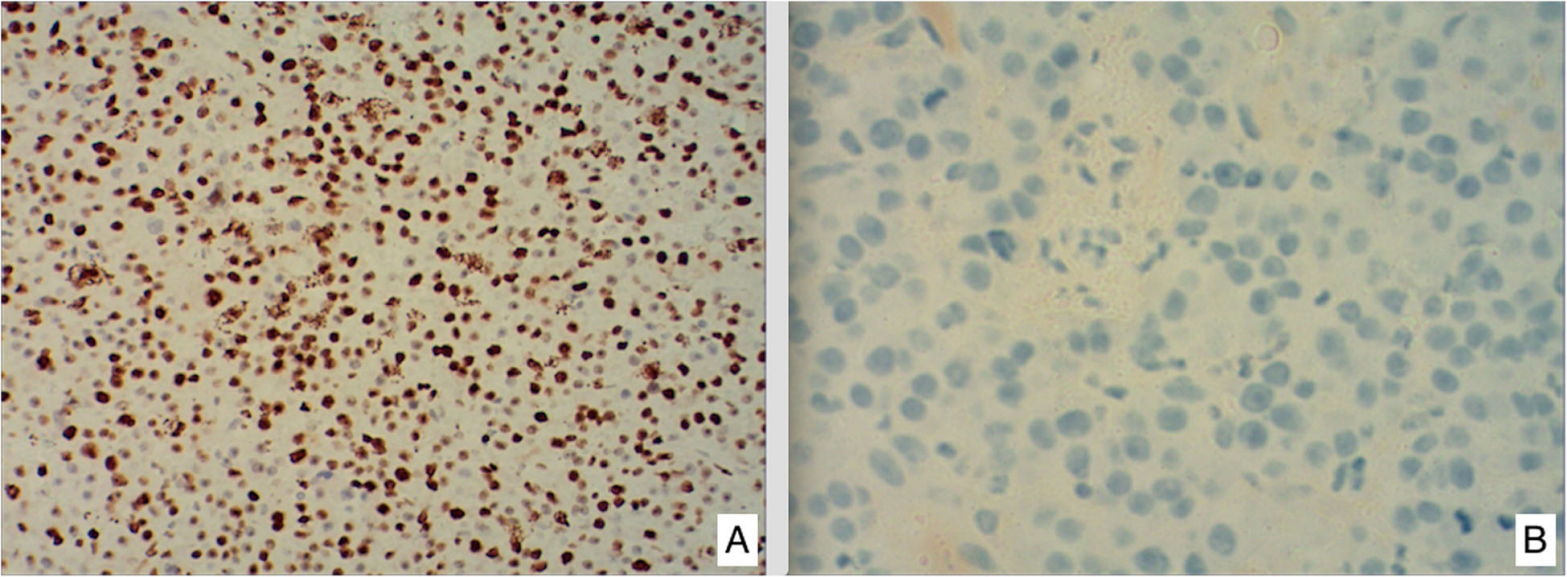
Negative marking results in CDX-2 (**a**) and CK-20 (**b**), showing enterogenesis is not supported

At this admission, abdominal enhanced CT revealed a thickened sigmoid wall with adjacent nodules (Fig. 1), as well as a nodule in the right lobe of the liver. The patient’s AFP level was 2.10 × 10^4^ ng/ml which was extremely high during his whole history. Chest CT scan showed a nodule in the dorsal segment of the lower lobe of the left lung, 13 mm in diameter, which was first considered to be a metastasis. The patient received enterolysis and Hartmann procedure (tumor resection and descending colostomy) on May 17, 2019. During the operation, we found a large endogenic mass of the colon, a relatively smooth serous layer (Fig. 2a, b) and clean intraperitoneal conditions. The operation was successful, and the intraoperative bleeding volume was about 20 ml. The postoperative pathological diagnosis was poorly differentiated adenocarcinoma accumulating in the whole intestinal wall, positive in vascular thrombosis, negative in nerve invasion, and no metastasis was found in 17 peri-intestinal lymph nodes. The distal, proximal, and circumferential cutting edges were all negative, while two cancerous nodules were observed (Figs. 3 and 4). Further immunohistochemical testing clarified the hepatocellular origin from the positive expression of heat shock protein 70 (HSP70), AFP and B-catenin, and negative expression of caudal-related homeobox transcription factor 2 (CDX-2) and eytokertin-20 (CK-20), with antigen Ki67 (Ki-67(+)) 80% (Figs. 5 and 6). The patient had recovered smoothly without any complications and was discharged on May 27. He continued taking toripalimab injection every 3 weeks since June 8, and TACE was performed again on June 12 to cure the nodule in the liver. Entecavir tablets were prescribed to anti-HBV virus by a hepatologist synchronously. Besides monthly blood tests of the tumor marker and HBV-DNA copy, he was also told to have enhanced abdominal CT and liver MRI every 3 months. The recent follow-up in August 2019 showed no signs of recurrence or metastasis in both the abdomen and liver. The AFP level also fell to 1.33 × 10^3^ng/ml from 2.10 × 10^4^ng/ml. Chest CT scan indicated that the pulmonary nodule was smaller than before.

## Discussion

HCC is a highly malignant tumor with a high rate of metastasis. Extrahepatic metastases are less frequent than intrahepatic ones with the incidence reported between 13.5–42% of metastasis cases. Uka et al. [2] investigated 151 patients with extrahepatic metastases from 995 HCC patients followed up regularly, and found that extrahepatic metastases were most frequently seen in the lungs (47%), lymph nodes (45%), bones (37%), and adrenal glands (12%), and most of them were even asymptomatic, which could only be found by autopsy. The ratio of extrahepatic metastases to the GI tract was extremely low (0.7%), only one case of colon metastasis detected from autopsy. Lin et al. [3] found 11 GI tract metastases of 2237 HCC patients at their hospital during more than 9 years. Chen et al. [4] collected 396 patients with HCC between the year 1982 to 1988 in their hospital, and identified 8 cases (2%) with secondary involvement of the GI tract, but no colon metastasis case was included (3 stomach, 4 duodenum, 1 jejunum, and 1 unknown). Terada et al. [5] found only 1 case of colon involvement as unusual extrahepatic metastatic sites among 52 extrahepatic metastasis cases in both autopsy (*n* = 31) and surgery (*n* = 21). Some studies also showed a low rate of GI involvement from HCC of about 4–12%, though not so low as in the previous ones [6, 7]. Case report covering GI involvement from HCC was limited (Table 1). Among all these cases, the most possible metastatic way is direct invasion, so upper GI is its favorite target organ, such as the stomach and duodenum. Chen et al. [4] also warned the doctors of direct invasion of the GI tract after regional treatment such as transcatheter arterial embolization (TAE) because of inflammatory response and adhesion to the liver or tumor capsule. Tumor thrombi will also result in metastasis to the stomach via the portal system. Upper GI bleeding is the most common symptom, and some massive bleeding may be followed by coma which can be regarded as a terminal episode of the disease [22]. Besides GI bleeding, abdominal pain, intussusception, and intestinal obstruction are also common manifestations in these patients.

**Table 1:** Summary of patients with GI metastasis from HCC in case report literature

Study (year)	Age	Sex	Underlying liver disease	Symptom	Site of metastasis	Interval (year)	Previous treatment	Survival period
Zhu et al. (2016) [8]	47	M	Hepatitis B	Positive in FOBT*	Transverse colon	5	Surgery, TAE^◇^, TACE^◆^	Over 12 months
Yoo et al. (2010) [9]	47	M	Hepatitis B	Abdominal pain	Sigmoid colon	1.5	TACE	Over 4 months
Igawa et al. (2013) [10]	60	M	Liver cirrhosis type B	Melena and anemia	Ileum	Unknown	Chemotherapy	2 months
Huang et al. (2011) [11]	57	F	Hepatitis C	Bloody stool	Rectum	1.5	Surgery	Unknown
Nozaki et al. (2008) [12]	69	M	Unknown	Abdominal pain and hematochezia	Ascending colon	2	Surgery	Less than 1 month
Tapuria et al. (2007) [13]	67	M	Autoimmune chronic active hepatitis	Bleeding per rectum, anemia	Proximal ascending colon	10	Immunosuppression	A few months
Kohli et al. (2014) [14]	50	F	Cryptogenic cirrhosis	Hematochezia	Splenic flexure	4	Orthotopic liver transplant, radioembolization	Unknown
Ou et al. (2014) [15]	62	M	Hepatitis B	Tenesmus	Ascending colon and rectum	3	Surgery, RFA^☆^, PEI^★^, CyberKnife stereotactic radiosurgery, TACE	1 month
Kanazawa et al. (2018) [16]	76	M	Alcoholic cirrhosis	Lightheadedness and melena	Upper jejunum and 5 cm from the lesion on the anal side	Unknown	Surgery, TACE, sorafenib	2 weeks
Iwaki et al. (2008) [17]	60	M	Acute hepatitis, hepatitis C, liver cirrhosis	Asymptomatic	Jejunum	4	Surgery, RFA, TACE	Unknown
Yang et al. (1987) [18]	31	M	Unknown	Anemia	Proximal jejunum	1.5	Surgery	Unknown
Cosenza et al. (1999) [19]	82	F	Hepatitis C	Weakness, fatigue, bright red blood per rectum	Ascending colon	4	Surgery, chemotherapy	Unknown
Kim et al. (1999) [20]	65	M	Hepatitis B	Periumbilical pain	Jejunum	3	None	Unknown
Hirashita et al. (1999) [21]	79	M	Hepatitis C	Epigastralgia	Transverse colon	1.5	TACE	6 months
	69	M	Hepatitis C	Melena and abdominal distension	Hepatic flexure and diaphragm	2.5	RFA and TACE	1 month

*FOBT, fecal occult blood test

^◇^TAE, transcatheter arterial embolization

^◆^TACE, transcatheter arterial angiography and chemoembolization

^☆^RFA, radiofrequency ablation

^★^,PEI, percutaneous ethanol injection

Compared to the upper GI tract, the colon and the rectum are much more rare locations for HCC metastases owing to the indirect position to the liver and upper stream of venous backflow. Ou et al. [15] searched MEDLINE database and listed 8 reports including patients with colonic metastasis from HCC in recent years and revealed that ascending and transverse colons were the most frequent sites in direct invasion as they were more adjacent to the primary region than any other parts of the colon. Intraperitoneal implantation is the most possible way for this kind of metastasis. That means the metastatic tumor will first deposit in the serous membrane surrounding the intestinal wall, and the mucous membrane is the last layer for the tumor to spread. The mass may compress or encase the intestines when it grows. So it is unusual to see the integrity lesion inside the intestinal cavity by colonoscopy. Yoo et al. [9] once reported such a case, and no tumor could be seen through colonoscopy but a bulging contoured mass. It was different in our case. At first, we did not realize the tumor in the sigmoid colon to be an extrahepatic metastasis from HCC because of the obvious mass growing in the colon wall which could be easily recognized by colonoscopy. During the operation, we still believed it to be a typical colon cancer because of the relatively smooth serous layer and clean intraperitoneal conditions. The tumor grew as large as 90 × 70 × 25 mm, and the way of metastasis remained unknown, neither from direct invasion nor from intraperitoneal implantation. Hematogenous metastasis was the only possible way, which might explain the source of the mass. Hu et al. [23] studied seven stomach involvement cases of 8267 HCC patients and found that patients with cirrhosis and/or portal vein thrombosis were more vulnerable to hematogenous pathway gastric metastasis when having TAE treatment because of portal hypertension. Kohli et al. [14] also reported a patient with cryptogenic cirrhosis suffering from splenic flexure metastasis from HCC after liver transplantation due to hematogenous metastasis. Our patient received TACE four times before sigmoid colon tumor resection, so we boldly hypothesized that TACE might increase the portal venous pressure inducing the blood to flow backward to the colon through inferior mesenteric vein, which finally formed that huge metastatic lesion.

HCC patients with advanced primary tumor stage (T3, T4) are at high risk of developing extrahepatic metastases, and on the other hand, most GI metastases are found in patients with advanced stage HCC [2, 9]. Though there is no standard guideline, surgical intervention should be practiced to relieve the symptoms or treat life-threatening GI metastases under the circumstances that the patient has good physical condition and adequate hepatic reserve. According to Fujii et al. [24], the median survival time of HCC patients with GI tract invasion treated by surgical resection is longer than those who receive nonsurgical and supportive treatment, but this result probably has bias because the latter ones may give up surgery on account of their late disease staging or poor physical condition. Multivariate analysis revealed that younger age, normal alpha fetoprotein, single site of extrahepatic disease, local treatment to the primary tumor, and surgery to the metastatic disease were associated with better overall survival and liver cancer-specific survival [25]. Though surgery, to some extent, can improve the survival of HCC patients with extrahepatic metastases, the prognosis of these patients is still poor. The median survival of HCC patients with colonic metastasis was only 2.5 months [21], which was even shorter than 8.1 months in patients with other extrahepatic metastasis [26]. GI bleeding is the most common complication, and in some serious cases may lead to death directly. Bowel obstruction is another life-threatening consequence such as the one in our case. For sure, surgery cannot cure the disease, but it may relieve the symptoms and prolong overall survival.

## Conclusions

Patients who have had a history of HCC with high AFP level initially diagnosed with colorectal cancer should be considered extrahepatic metastases from HCC as well. Direct invasion, intraperitoneal implantation, and hematogenous metastasis are the most possible ways for the tumor to spread. TACE/TAE will raise the portal venous pressure, which may cause retrograde hematogenous metastases to the downstream colon. Though HCC patients with colon metastasis have very poor prognosis, surgery is an effective way to relieve the symptoms and prolong overall survival.

## Data Availability

All data and material are fully available without restriction.

## Abbreviations

HCC: Hepatocellular carcinoma
CT: Computerized tomography
AFP: Alpha-fetoprotein
GI: Gastrointestinal
MRI: Magnetic resonance imaging
TACE: Transcatheter arterial chemoembolization
HBV: Hepatitis B virus
CEA: Carcinoembryonic antigen
PRFA: Percutaneous radiofrequency ablation
TAE: Transhepatic arterial embolization
